# Descending Pain Modulation in Fibromyalgia: A Short Review of Mechanisms and Biomarkers

**DOI:** 10.3390/diagnostics15212702

**Published:** 2025-10-25

**Authors:** Bruno Daniel Carneiro, Sandra Torres, José Tiago Costa-Pereira, Daniel Humberto Pozza, Isaura Tavares

**Affiliations:** 1 Unit of Experimental Biology, Department of Biomedicine, Faculty of Medicine, University of Porto, 4200-319 Porto, Portugal; bcarneiro@med.up.pt (B.D.C.); jcostapereira@fcna.up.pt (J.T.C.-P.); dhpozza@gmail.com (D.H.P.); 2 Rheumatology Service, Unidade Local de Saúde do Alto Minho, Hospital Conde de Bertiandos, 4990-078 Ponte de Lima, Portugal; 3 Unidade de Saúde Familiar São João, Unidade Local de Saúde de Entre Douro e Vouga, 3700-298 São João da Madeira, Portugal; sandra.torres.med@gmail.com; 4 Faculty of Nutrition and Food Sciences, University of Porto, 4150-180 Porto, Portugal; 5 Institute for Research and Innovation in Health and IBMC (i3S), University of Porto, 4200-135 Porto, Portugal

**Keywords:** nociplastic pain, fibromyalgia, chronic pain, central sensitization, descending pain modulation, biomarkers

## Abstract

Fibromyalgia is a prevalent chronic pain disorder characterized by widespread musculoskeletal pain, fatigue, cognitive dysfunction, and sleep disturbances, with high impact in quality of life. Despite extensive research, the pathophysiological mechanisms of fibromyalgia remain partially understood, complicating the diagnosis and treatment. Some evidence underscores the central role of abnormal pain processing, particularly central sensitization and defective descending pain modulation pathways. This review synthesizes and discusses current findings on the neurobiological underpinnings of pain in fibromyalgia, with focus on descending inhibitory control mechanisms and on the role of biomarkers. We integrate data from neurochemical, neuroimaging, and clinical studies to explain how impaired descending modulation contributes to enhanced pain sensitivity and discuss the putative biomarkers associated with changes in descending modulation. A better understanding of descending pain modulation dysfunction in fibromyalgia and related biomarkers is crucial for improving clinical outcomes and developing novel and more effective treatments.

## 1. Introduction

Fibromyalgia is a complex, multifactorial syndrome affecting approximately 2–4% of the general population worldwide, with a striking female predominance [1,2]. Clinically characterized by chronic widespread musculoskeletal pain, fatigue, sleep disturbances, cognitive dysfunction (“fibro fog”), and mood disorders, fibromyalgia implicates significant challenges for both patients and clinicians due to its heterogeneous presentation and poorly understood etiology [3,4,5,6]. The American College of Rheumatology (ACR) diagnostic criteria, published in 2010 and updated in 2016, emphasize symptom severity and widespread pain but lack objective biomarkers, underscoring the need for deeper mechanistic insights [7].

The burden of fibromyalgia extends beyond physical symptoms, significantly impacting daily functioning, psychosocial well-being, and health care utilization [8,9,10]. Patients experience comorbid conditions such as irritable bowel syndrome, temporomandibular disorders, and migraine, highlighting the systemic nature of fibromyalgia [11]. This complexity demands a comprehensive understanding of the underlying pathophysiology to guide diagnosis, prognosis, and personalized treatment strategies.

**Figure 1:**
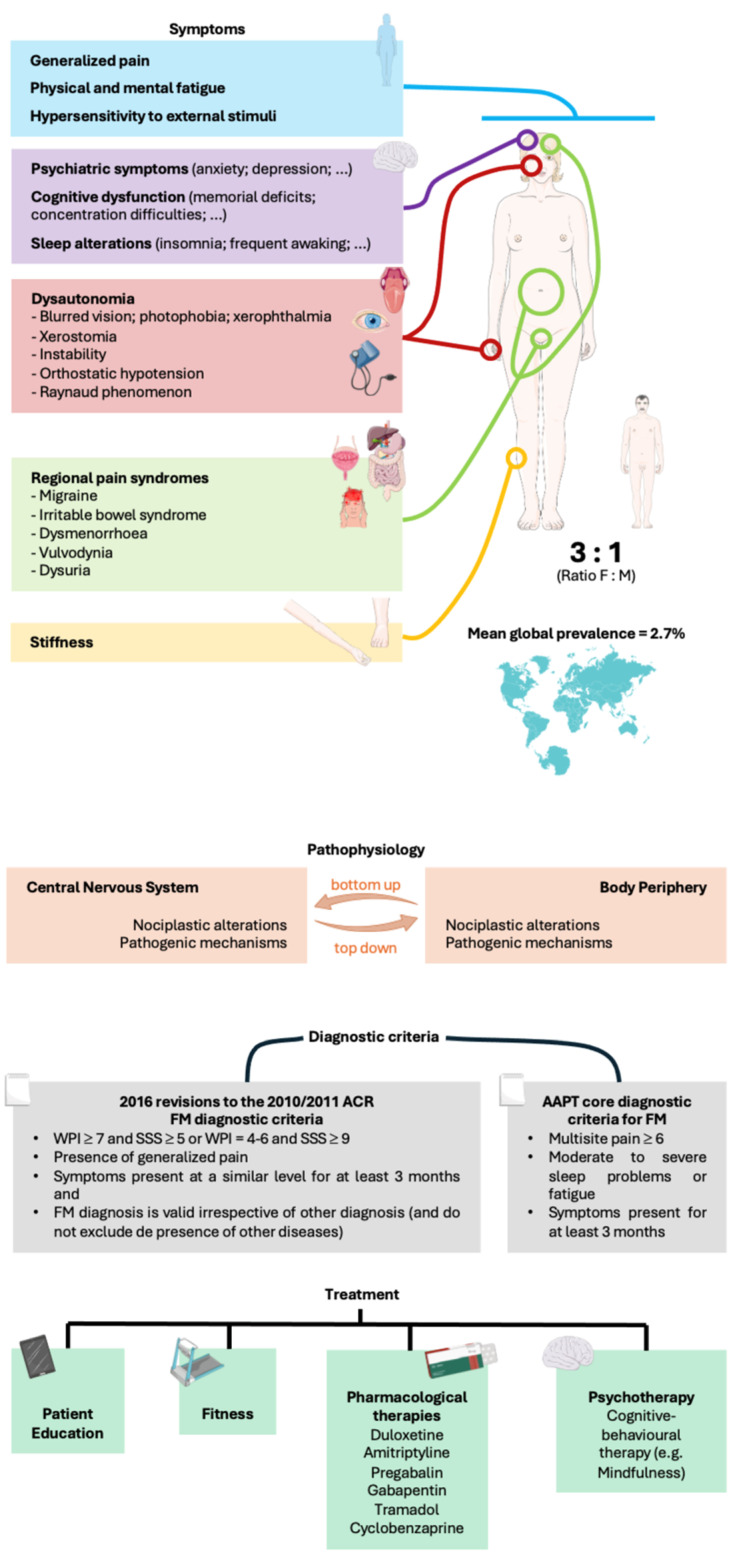
Infogram about fibromyalgia. AAPT, ACTTION (Addiction Clinical Trial Translations Innovations Opportunities and Networks)-APS (American Pain Society) Pain Taxonomy; ACR, American College of Rheumatology; F, female; FM, fibromyalgia; M, male; SSS, symptom severity score; WPI, widespread pain index. Parts of the figure were drawn using pictures from Servier Medical Art by Servier that is licensed under Attribution 4.0 International.

Figure 1 summarizes the epidemiology, symptoms, pathophysiology, diagnostic criteria and treatment of fibromyalgia.

Over the past two decades, accumulating evidence has shifted the conceptual framework of fibromyalgia from a purely peripheral nervous system and musculoskeletal disorder, with some studies suggesting that the disease is associated with small fiber neuropathy [12,13,14,15] and others suggesting that fibromyalgia patients exhibited greater variability in muscle fiber size and altered fiber size distribution [16], to a centralized pain syndrome involving aberrant central nervous system (CNS) processing [1,17,18]. In fact, central sensitization (CS), a neuroplastic phenomenon characterized by amplified responsiveness of nociceptive neurons in the spinal cord and brain, is considered a landmark feature driving enhanced pain perception in fibromyalgia [19,20]. Moreover, dysfunction of the descending pain modulatory system (DPMS), which normally balances between inhibitory and facilitatory control, but exerting inhibition in acute pain, has emerged as a critical contributor to persistent pain and symptom amplification in fibromyalgia [21,22,23].

Descending pain modulation involves complex neural circuits spanning cortical, subcortical, and brainstem regions, including the prefrontal cortex (PFC), anterior cingulate cortex (ACC), periaqueductal gray (PAG), and rostral ventromedial medulla (RVM) [24]. These pathways utilize endogenous neurotransmitters, namely serotonin (5-HT), noradrenaline (NA), dopamine (D), and opioids, to suppress or facilitate pain signals at the dorsal horn level [25]. Dysfunction in these systems can activate pain facilitation and exacerbate clinical symptoms [26].

Despite advances, the precise mechanisms by which descending pain modulation is impaired in fibromyalgia remain incompletely elucidated. Neuroimaging studies revealed altered brain connectivity and functional changes within DPMS nodes, while neurochemical investigations highlight imbalances in monoaminergic and glutamatergic neurotransmission [27,28,29,30]. Furthermore, a recent multimodal neuroimaging study recruiting 40,000 participants, showed that fibromyalgia was strongly associated with relevant changes in subcortical brain structures involved in descending pain modulatory pathways, namely structural connectivity between PAG and amygdala, as well as between PAG and hypothalamus [31]. Identifying reliable and specific biomarkers associated with this impaired pain inhibition is pivotal for advancing both the pathophysiological understanding and clinical translation.

Given the increasing prevalence of fibromyalgia and its substantial impact on public health, as well as the need for high-quality and objective syntheses of current knowledge, a comprehensive and up-to-date review of the mechanisms underlying pain and dysfunction in descending pain modulation, along with potential biomarkers, is both timely and essential. Such insights will enhance our understanding of diagnostic biomarkers and support the development of novel therapeutic targets tailored to the neurobiological profiles of individual patients.

Therefore, this review aims to provide a synthetic overview of the current scientific evidence on the neurobiological mechanisms of pain in fibromyalgia, with focus on the role of descending pain modulation and the role of biomarkers.

## 2. Methods

This review includes evidence from the last 3 decades, obtained from the MEDLINE/PubMed and Scopus scientific databases, without any restriction. We included all the articles that summarized evidence about the theme, namely by searching for works using the terms “fibromyalgia”, “pain”, “biomarkers”, “mechanisms” and “descending pain modulation”, after a critical and qualitative analysis of all articles by all the authors. We critically appraise neurophysiological, neurochemical, neuroimaging, and clinical data to delineate how abnormalities in pain inhibitory pathways contribute to symptomatology. Finally, we evaluate the clinical implications and emerging therapeutic strategies targeting these pathways.

## 3. Pathophysiology of Pain in Fibromyalgia

Fibromyalgia pain is multifactorial and appears to result from complex interactions between peripheral and central nociceptive mechanisms. Although some peripheral tissue abnormalities (e.g., muscle microtrauma or inflammation) may initiate nociceptive input, persistent widespread pain in fibromyalgia predominantly reflects CNS amplification and altered pain processing [32,33,34].

Central sensitization increases excitability and synaptic efficacy of nociceptive neurons in the dorsal horn and higher centers leading to hyperalgesia (exaggerated pain responses to stimuli) and allodynia (pain elicited by normally innocuous stimuli) [35]. Impaired descending pain inhibition leads to dysfunction of brainstem and cortical circuits responsible for endogenous analgesia, resulting in insufficient suppression of nociceptive transmission [36]. Moreover, altered levels and receptor function of key neurotransmitters (e.g., 5-HT and NA) disrupt both pain modulation and affective components of pain [37,38]. Microglia and astrocyte activation in the CNS release pro-inflammatory cytokines and chemokines, which sensitize nociceptive neurons and disrupt neural circuits [39]. Reductions in gray matter volume and altered functional connectivity in pain-processing and modulatory regions further contribute to persistent pain and cognitive symptoms [40,41]. Together, these factors establish a maladaptive pain state in fibromyalgia, characterized by chronic widespread pain, fatigue, and cognitive disturbances.

### 3.1. Central Sensitization in Fibromyalgia

Central sensitization seems to be a key mechanism underpinning enhanced pain sensitivity in fibromyalgia. It involves increased responsiveness of nociceptive neurons in the spinal dorsal horn and brain to peripheral stimuli [42]. The mechanisms associated with CS are diverse. There are enhanced excitatory synaptic transmission mediated by increased glutamate (Glu) release and N-methyl-D-aspartate (NMDA) receptor activation [43], increased levels of excitatory neuropeptides such as substance P (SP) and calcitonin gene-related peptide (CGRP) [44] and long-term neuroplastic changes, including altered synaptic strength and receptor expression, in pain-related brain regions such as the insula, ACC, thalamus, and PFC [45,46].

Clinical correlates of CS include widespread pain, tenderness, allodynia, and secondary hyperalgesia. Quantitative sensory testing (QST) and functional neuroimaging support the presence of CS in fibromyalgia patients [47,48]. These patients experience an exaggerated increase in perceived pain intensity in response to rapidly repeated noxious stimuli—a phenomenon known as temporal summation of pain [49,50]. However, there are also studies suggesting that fibromyalgia patients are hyperalgesic in spite of a normal modulation of the transmission of nociceptive input [51,52].

The increased pain sensitivity in fibromyalgia is part of complex hypersensitivity to sensory stimuli in general, including visual, auditory, and olfactory inputs. In fact, fibromyalgia patients have exhibited increased brain responses not only to the pain onset but also its offset [53], as well as hypersensitivity to sound and heat [54].

### 3.2. Dysfunction of Descending Pain Modulation in Fibromyalgia

The DPMS regulates nociceptive transmission through both inhibitory and facilitatory influences originating from cortical and brainstem regions [25]. Its principal components include: (1) the PFC and ACC, which are involved in the cognitive and emotional modulation of pain [55]; (2) the PAG, a midbrain region critical for initiating descending inhibition via endogenous opioids [56]; (3) RVM, which exerts a bidirectional control of nociception by its resident on- and off-cells that facilitate or inhibit spinal nociceptive neurons, respectively [57]; (4) the locus coeruleus (LC), which provides noradrenergic descending projections [58]; and (5) the nucleus raphe magnus (NRM), a key source of serotonergic descending fibers [26].

In healthy individuals, the DPMS may modulates pain signals via neurotransmitters such as 5-HT, NA, D, and endogenous opioids, facilitating adaptive responses to noxious stimuli [59]. Multiple changes in how pain is transmitted and modulated, namely those involving key neurotransmitters, are thought to disrupt the balance between pain-inhibiting and pain-facilitating pathways [59]. This imbalance contributes to the chronification of pain and CS [59].

Numerous studies demonstrate impaired descending inhibition in fibromyalgia. In fact, fibromyalgia patients consistently show reduced descending pain modulation efficiency compared to healthy controls, indicating defective endogenous pain inhibition [50,60,61]. Conditioned pain modulation (CPM), the human correlate of diffuse noxious inhibitory controls (DNIC), reflects DPMS efficacy and is measurable through experimental paradigms [62]. In CPM testing a painful conditioning stimulus is applied in one part of body, which leads to a reduction in pain perception from another, separate painful stimulus applied elsewhere, often on the opposite side. This response illustrates the CNS’s ability to suppress pain signals in one region when another painful input is present, demonstrate an endogenous pain inhibition mechanism [63]. Similarly to other chronic pain conditions, research has indicated that CPM tends to be less effective in fibromyalgia patients [50,64,65,66,67].

Furthermore, functional magnetic resonance imaging (fMRI) studies reveal reduced activity and altered connectivity in DPMS structures, such as diminished PAG and ACC engagement during pain modulation tasks [68,69] and positron emission tomography (PET) imaging shows altered opioid receptor availability in DPMS regions in fibromyalgia [70]. Neurochemical studies reveal decreased serotonergic and noradrenergic tone, impairing descending inhibitory signaling [70,71]. This dysfunction disrupts the balance between pain facilitation and inhibition, promoting persistent pain and symptom exacerbation. Reduced levels of 5-HT and 5-HT-receptor dysfunction decrease descending inhibition and contribute to affective symptoms [72]. Deficits in signaling associated with NA impair pain inhibition and arousal regulation [73]. Altered dopaminergic neurotransmission, involving D, affects reward processing, fatigue, and pain perception [74]. Increased activity of Glu pathways enhances excitatory signaling and CS [33]. Elevated cerebrospinal fluid (CSF) concentrations of SP correlate with pain intensity [75]. The imbalance of these neurotransmitters represents the basis for pharmacologic interventions targeting serotonin-noradrenaline reuptake inhibitors (SNRIs), D agonists, and Glu modulators [76].

An abnormal resting-state functional connectivity of the PAG was found in fibromyalgia patients [77] and these changes result in impaired descending pain inhibition. Patients with fibromyalgia were also found to have altered functional connectivity with the default mode network (a region active when the brain is at rest) and the insula [78]. Altered hub topology within the insula was associated with clinical pain intensity. In line with this, the organization of intrinsic functional brain hubs or communities in patients with fibromyalgia demonstrate decreased neural stability.

#### Neuroinflammation and Glial Activation

Neuroinflammation involves the activation of the immune cells in the CNS, namely microglia and astrocytes, and subsequent release of pro-inflammatory mediators. This phenomenon plays a significant role in dysregulation of the DPMS [79]. Emerging research implicates neuroinflammatory processes as a critical feature in fibromyalgia pathogenesis (reviewed by Findeisen and collaborators [80]). Activated microglia and astrocytes release cytokines, such interleukins (IL) 6 and 1β and tumor necrosis factor α, and chemokines, that sensitize nociceptive neurons and impair descending modulation [81,82]. Elevated inflammatory markers in CSF and blood have been reported in fibromyalgia [83,84]. Furthermore, recent studies utilizing PET-computed tomography brain imaging with the 18 kDa Translocator Protein (TSPO) binding have revealed a widespread cortical glial activation in fibromyalgia patients [85,86].

Neuroinflammation appears to be a key factor in maintaining CS and is also contribute to comorbid symptoms such as fatigue and cognitive dysfunction [87]. As a result, therapeutic approaches aimed at glial activation and reducing inflammation are currently considered as promising strategies in the management of fibromyalgia [88].

### 3.3. Brain Structural and Functional Changes

Advanced neuroimaging techniques reveal consistent structural and functional brain abnormalities in fibromyalgia such as: (1) gray matter volume reductions in pain-related regions including the insula, ACC, PFC, and hippocampus [89,90,91]; (2) altered white matter microstructure in pain pathways [92]; and (3) aberrant resting-state functional connectivity within the default mode, salience, and sensorimotor networks [33,93]. A recent study also suggests that individuals with fibromyalgia exhibited changed anticipatory pain processing, marked by reduced activation of the dorsolateral PFC and disrupted connectivity with pain processing brain regions, which may underlie their impaired ability to modulate pain expectations [94].

All these changes correlate with clinical symptom severity and cognitive dysfunction [95] and support the concept of fibromyalgia as a disorder of CNS involving both sensory and affective pain dimensions. Interestingly, multimodal brain imaging demonstrated that gray-matter volumes reduction in patients with fibromyalgia are correlated with the low levels of tissue water content, suggesting neuronal plasticity [96] which is in line with the concept of nociplastic pain.

## 4. Biomarkers

In fibromyalgia, diagnostic biomarkers may serve as critical tools for delineating pathophysiological mechanisms, stratifying patients into biologically relevant subgroups within this heterogenous pain condition and characterizing molecular and physiological changes associated with the treatment [97,98,99,100].

A widely accepted functional biomarker of descending pain inhibition is the previous referred CPM. In fibromyalgia, the CPM response is significantly diminished, supporting impaired top-down pain modulation and consistent with the concept of CS [62,101,102]. The degree of CPM dysfunction has been found to correlate with clinical symptoms severity and may serve as a predictive marker for treatment response [103].

Advanced neuroimaging techniques including fMRI, PET, and single photon emission computed tomography (SPECT) have demonstrated altered connectivity and increased activity in regions associated with pain perception and modulation [69,104]. In addition, PET imaging using TSPO ligands, such as PBR28 and DPA-714, has revealed increased microglial activation in cortical regions suggesting neuroinflammation as a central mechanism [86,105]. The neuroinflammatory changes were positively correlated with pain intensity and fatigue levels in fibromyalgia, positioning TSPO-PET as a candidate biomarker. Fibromyalgia has also been associated with a low-grade pro-inflammatory profile with elevated levels of IL-6 and IL-8 being repeatedly found in the serum and plasma of fibromyalgia patients, associated with pain intensity, fatigue, and cognitive symptoms [33,106]. Anti-inflammatory cytokines, such as IL-10, may be elevated as a compensatory response [106,107].

Numerous neurochemical alterations associated with DPMS dysfunction have been identified in fibromyalgia with higher potential to be biomarker: (1) elevated serum fluid levels of brain-derived neurotrophic factor (BDNF) have been found in fibromyalgia patients [108]; (2) fibromyalgia patients exhibit higher CSF levels of SP [75]; (3) reduced levels of 5-HT, D, and NA in the CNS and peripheral tissues reflect impaired descending inhibitory neurotransmission [109]; (4) spectroscopy imaging studies employing ^1^H-MRI (proton nuclear magnetic resonance) have demonstrated higher levels of Glu in pain-processing brain regions of fibromyalgia patients, suggesting Glu as a useful biomarker of pain in in this population [33,110,111].

Epigenetic modifications and non-coding ribonucleic acid (RNA) may regulate genes involved in pain modulation with methylation changes being observed in genes involved in monoaminergic neurotransmission (e.g., solute carrier family 6 member 4—SCL6A4), Glu receptors (e.g., glutamate metabotropic receptor 6—GRM6), and neuroplasticity (e.g., BDNF) [112]. Several microRNA (miR), such as miR-145-5p, miR-23a-3p and miR-21 have been reported to be dysregulated in fibromyalgia and correlated with pain intensity and fatigue. These may serve as non-invasive biomarkers with potential regulatory roles in DPMS [113].

Patients with fibromyalgia often exhibit autonomic imbalance along with dysfunction of the hypothalamic–pituitary–adrenal axis dysfunction, both of which are linked to impaired stress response and pain regulation. Biomarkers in this domain include low cortisol levels [114,115].

Fibromyalgia patients also have higher levels of C-reactive protein, and these higher levels could be associated with higher symptom severity [116,117]. However, a systematic review did not fully support the relation between the levels of C-reactive protein and symptom severity [118].

Table 1 presents a synthetic integration of biomarkers with descending pain modulation in fibromyalgia.

## 5. Clinical Implications and Therapeutic Perspectives

Understanding descending pain modulation deficits has direct clinical relevance. Regarding pharmacological therapies, SNRIs, such as duloxetine, restore monoaminergic tone and improve pain and mood [119], gabapentinoids modulate excitatory neurotransmission [120] and low-dose naltrexone may reduce neuroinflammation and enhance endogenous opioid activity [121]. Surprisingly, a recent randomized clinical trial suggested that the effects of low-dose naltrexone in fibromyalgia patients may be attributed to a decline in CPM within placebo group than to a robust analgesic effect of the drug [122], underscoring the complexity of fibromyalgia pathophysiology and its treatment.

On the other hand, non-pharmacological therapies, such as cognitive-behavioral therapy (CBT) and mind–body interventions (namely mindfulness-based interventions), and aerobic exercise, improve DPMS function and neuroplasticity [123,124]. Neuromodulation, namely transcranial magnetic stimulation (TMS) and transcranial direct current stimulation (tDCS) show promise in enhancing descending inhibition [125,126,127].

The role of microbiome in fibromyalgia cannot be overlooked, since recent studies have highlighted its involvement, particularly in modulation of pain during this pathology [128,129,130]. Therefore, these findings suggest that nutritional interventions may offer a promising non-pharmacological therapeutic strategy with the potential to positively fibromyalgia management [131,132].

A better knowledge about biomarkers of DPMS function and neuroinflammation may enable tailored therapies [133].

## 6. Concluding Remarks

Fibromyalgia represents a complex, multifactorial syndrome characterized by chronic widespread pain, fatigue, cognitive dysfunction, and a host of somatic symptoms. Despite decades of research, its precise etiology remains elusive, with current evidence supporting a CS paradigm involving aberrant pain processing and neuroinflammation. The literature consistently highlights altered CNS function, including dysregulation of neurotransmitters and changes in brain connectivity patterns that correlate with symptom severity [1,33,69]. These neurobiological alterations offer a plausible mechanistic explanation for the hallmark symptoms of fibromyalgia and underscore the syndrome’s classification as a disorder of central pain amplification rather than purely peripheral pathology.

**Figure 2:**
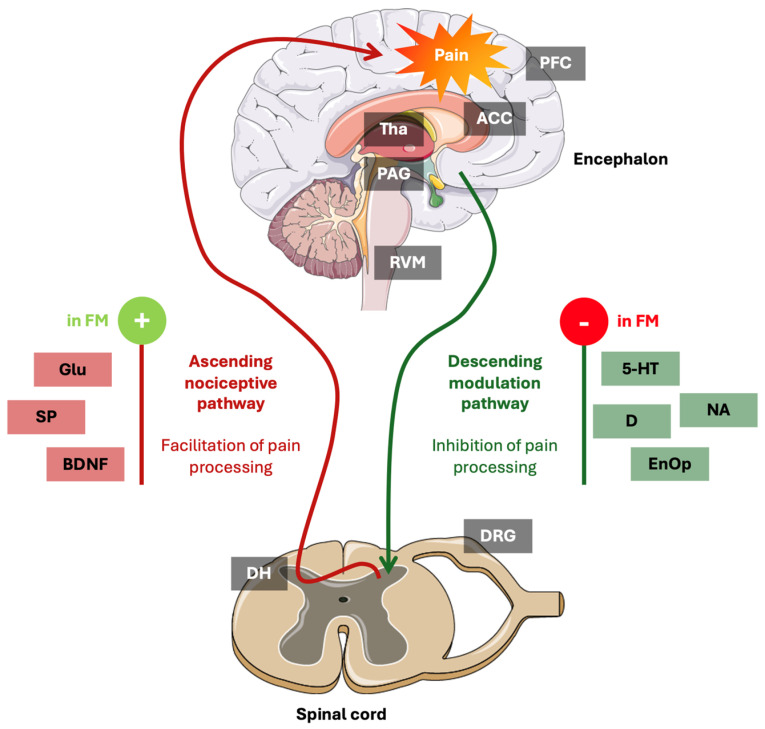
Infogram about pain pathways and neurotransmitters in fibromyalgia. ACC, anterior cingulate cortex; BDNF, brain-derived neurotrophic factor; D, dopamine; DH, dorsal horn; DRG, dorsal root ganglion; EnOp, endogenous opioids; FM, fibromyalgia; Glu, glutamate; 5-HT, serotonin; NA, noradrenaline; PAG, periaqueductal gray; PFC, prefrontal cortex; RVM, rostral ventromedial medulla; SP, substance P; Tha, thalamus. Parts of the figure were drawn using pictures from Servier Medical Art by Servier that is licensed under Attribution 4.0 International.

In Figure 2, we summarized the pain pathways and neurotransmitters in fibromyalgia.

However, the heterogeneity of clinical presentations and overlap with other central sensitivity syndromes complicates diagnosis and management. Under- and misdiagnosis remain challenges, often leading to delayed treatment and reduced quality of life [4]. This calls for continued refinement of diagnostic criteria and the development of objective biomarkers, which remain an unmet need despite promising findings in neuroimaging and CSF inflammatory markers [21,95].

Therapeutic approaches have evolved to target the underlying neurochemical imbalances and CS processes. Pharmacologic interventions such as duloxetine and pregabalin, which modulate 5-HT and NA pathways, provide symptomatic relief for some patients, but efficacy is highly variable [119,120]. Interestingly in a recent study, carried out in rats, was outline the antidepressant aptitude of milnacipran and vanillin through activating Wnt/β-catenin signaling in the hippocampus in reserpine-induced fibromyalgia [134]. Non-pharmacological strategies, including CBT and graded exercise, address the biopsychosocial aspects and demonstrate substantial benefit, emphasizing the importance of multidisciplinary care [123,124]. In fact, international recommendations for the treatment of fibromyalgia highlight the non-pharmacological approaches as the first line therapy, establishing three essential components: patient education; CBT; and exercise [1,135,136,137,138].

Other emerging therapies such as low-dose naltrexone, neuromodulation techniques and dietary intervention are promising but require further rigorous trials [88,125,129].

Future research should focus on unraveling the interplay between genetic predisposition, neuroimmune interactions, and environmental triggers to better stratify patients and personalize treatment. Advances in neuroimaging and molecular biomarkers may allow clinicians to move beyond symptom-based diagnosis toward precision medicine. Additionally, understanding the role of neuroinflammation and glial activation could open novel therapeutic avenues targeting immune-neural crosstalk in chronic pain syndromes. The presence of multiple biomarker domains in fibromyalgia reflecting impaired descending pain modulation provide a robust framework to objectively assess the status of the DPMS. These biomarkers hold promise not only for improving diagnostic accuracy but also for patient stratification, prognosis, and response prediction to mechanism-based treatments in fibromyalgia.

In this review, we highlighted that fibromyalgia is best conceptualized as a disorder of CNS dysregulation with significant biopsychosocial dimensions. While progress has been made in elucidating its pathophysiology and improving management, comprehensive, individualized treatment plans remain essential to address the syndrome’s complexity and improve patient outcomes, and for this it is urgent and essential to understand the role of descending modulation of pain in fibromyalgia and the role of the associated biomarkers.

In conclusion, fibromyalgia is a prototypical centralized pain syndrome driven by complex neurobiological mechanisms. CS and impaired descending pain modulation underlie the amplified pain experience. This dysregulation is further sustained by neurotransmitter imbalances, neuroinflammation, and brain structural and functional alterations. Advances in neurobiology and neuroimaging hold promise for increasing our understanding of fibromyalgia and offer new avenues for precision medicine approaches. Future research should focus on identifying more rigorous biomarkers, clarifying their role, and the development of targeted therapies that restore endogenous pain inhibitory mechanisms to alleviate the burden of fibromyalgia.

## Figures and Tables

**Table 1:** Integration of biomarkers with descending pain modulation in fibromyalgia.

Category	Example Biomarkers	Relation with Descending Pain Modulation
Functional	CPM	Allow direct measure of endogenous inhibitory system
Neuroimaging	TSPO-PET; fMRI	Indicators of neuroinflammation and cortical dysregulation
Neurochemical	BDNF; SP; 5-HT	Indicators of excitatory and inhibitory neurotransmission
Immunological	IL-6; IL-8; IL-10	Indicators of neuroimmune involvement and sensitization
Epigenetic	GRM6; BDNF; miR	Indicators of genetic regulation of DPMS
Hypothalamic–pituitary–adrenal axis control	Cortisol	Indicators of systemic dysregulation affecting pain

**Legend:** BDNF, brain-derived neurotrophic factor; CPM, conditioned pain modulation; DPMS, descending pain modulatory system; fMRI, functional magnetic resonance imaging; GRM6, glutamate metabotropic receptor 6; 5-HT, serotonin; IL, interleukin; miR, micro ribonucleic acid; SP, substance P; TSPO-PET, 18 kDa Translocator Protein-positron emission tomography.

## Data Availability

All data generated during this study are available upon reasonable request from the corresponding author.

## Abbreviations

The following abbreviations are used in this manuscript:

ACC	anterior cingulate cortex
ACR	American College of Rheumatology
BDNF	brain-derived neurotrophic factor
CBT	cognitive-behavioral therapy
CGRP	calcitonin gene-related peptide
CNS	central nervous system
CPM	conditioned pain modulation
CS	central sensitization
CSF	cerebrospinal fluid
D	dopamine
DNIC	diffuse noxious inhibitory controls
DPMS	descending pain modulatory system
FM	fibromyalgia
fMRI	functional magnetic resonance imaging
Glu	glutamate
GRM6	glutamate metabotropic receptor 6
^1^H-MRI	proton nuclear magnetic resonance
HPA	hypothalamic–pituitary–adrenal axis
HRV	heart rate variability
5-HT	serotonin
IL	interleukin
LC	locus coeruleus
miR	micro ribonucleic acid
NA	noradrenaline
NMDA	N-methyl-d-aspartate
NRM	nucleus raphe magnus
PAG	periaqueductal gray
PET	positron emission tomography
PFC	prefrontal cortex
QST	quantitative sensory testing
RNA	ribonucleic acid
RVM	rostral ventromedial medulla
SCL6A4	solute carrier family 6 member
SP	substance P
SNRIs	serotonin-norepinephrine reuptake inhibitors
SPECT	single photon emission computed tomography
tDCS	transcranial direct current stimulation
TMS	transcranial magnetic stimulation
TSPO	18 kDa Translocator Protein

## References

[1] Clauw D.J. (2014). Fibromyalgia: A Clinical Review. JAMA.

[2] Queiroz L.P. (2013). Worldwide epidemiology of fibromyalgia. Curr. Pain Headache Rep..

[3] Wolfe F., Clauw D.J., Fitzcharles M.A., Goldenberg D.L., Häuser W., Katz R.L., Mease P., Russell A.S., Russell I.J., Walitt B. (2016). 2016 Revisions to the 2010/2011 fibromyalgia diagnostic criteria. Semin. Arthritis Rheum..

[4] Häuser W., Ablin J., Fitzcharles M.A., Littlejohn G., Luciano J.V., Usui C., Walitt B. (2017). Fibromyalgia syndrome: Under-, over- and misdiagnosis. Clin. Exp. Rheumatol..

[5] Arnold L.M., Crofford L.J., Mease P.J., Burgess S.M., Palmer S.C., Abetz L., Martin S.A. (2012). Patient perspectives on the impact of fibromyalgia. Patient Prefer. Adherence.

[6] Häuser W., Jung E., Erbslöh-Möller B., Gesmann M., Kühn-Becker H., Petermann F., Tölle T., Uçeyler N., Sommer C. (2017). Fibromyalgia: Prevalence, characteristics, and treatment in clinical practice. Clin. Exp. Rheumatol..

[7] Wolfe F., Clauw D.J., Fitzcharles M.A., Goldenberg D.L., Häuser W., Katz R.L., Mease P., Russell A.S., Russell I.J., Winfield J.B. (2010). The American College of Rheumatology Preliminary Diagnostic Criteria for Fibromyalgia and Measurement of Symptom Severity. Arthritis Care Res..

[8] White K.P., Speechley M., Harth M., Ostbye T. (2000). Health-related quality of life in patients with fibromyalgia. J. Rheumatol..

[9] McBeth J., Macfarlane G.J. (2010). Health-related quality of life and socioeconomic status in fibromyalgia. Arthritis Care Res..

[10] Katz R.S. (1995). The impact of fibromyalgia on health status and employment. Arthritis Rheum..

[11] Wolfe F. (1990). The fibromyalgia syndrome: A critical review. J. Rheumatol..

[12] Üçeyler N., Zeller D., Kahn A.K., Kewenig S., Kittel-Schneider S., Schmid A., Casanova-Molla J., Reiners K., Sommer C. (2013). Small fibre pathology in patients with fibromyalgia syndrome. Brain.

[13] Oaklander A.L., Herzog Z.D., Downs H.M., Klein M.M. (2013). Objective evidence that small-fiber polyneuropathy underlies some illnesses currently labeled as fibromyalgia. Pain.

[14] Giannoccaro M.P., Donadio V., Incensi A., Avoni P., Liguori R. (2014). Small nerve fiber involvement in patients referred for fibromyalgia. Muscle Nerve.

[15] Caro X.J., Winter E.F. (2014). Evidence of abnormal epidermal nerve fiber density in fibromyalgia: Clinical and immunologic implications. Arthritis Rheumatol..

[16] Srikuea R., Symons T.B., Long D.E., Lee J.D., Shang Y., Chomentowski P.J., Yu G., Crofford L.J., Peterson C.A. (2013). Association of fibromyalgia with altered skeletal muscle characteristics which may contribute to postexertional fatigue in postmenopausal women. Arthritis Rheumatol..

[17] Yunus M.B. (2008). Central sensitivity syndromes: A new paradigm and group nosology for fibromyalgia and overlapping conditions. Arthritis Rheum..

[18] Staud R. (2012). Peripheral and central mechanisms of fatigue in inflammatory and noninflammatory rheumatic diseases. Curr. Rheumatol. Rep..

[19] Woolf C.J. (2011). Central sensitization: Implications for the diagnosis and treatment of pain. Pain.

[20] Mezhov V., Guymer E., Littlejohn G. (2021). Central sensitivity and fibromyalgia. Intern. Med. J..

[21] Harris R.E., Clauw D.J., Scott D.J., McLean S.A., Gracely R.H., Zubieta J.K. (2007). Decreased central mu-opioid receptor availability in fibromyalgia. J. Neurosci..

[22] Jensen K.B. (2016). Overlapping structural and functional brain changes in patients with long-term exposure to pain and opioid medication. Eur. J. Pain.

[23] Martikainen I.K. (2014). Dopaminergic and serotonergic mechanisms in fibromyalgia. Brain.

[24] Fields H.L. (2004). State-dependent opioid control of pain. Nat. Rev. Neurosci..

[25] Tracey I., Mantyh P.W. (2007). The cerebral signature for pain perception and its modulation. Neuron.

[26] Millan M.J. (2002). Descending control of pain. Prog. Neurobiol..

[27] Jensen K.B. (2015). Functional changes in endogenous pain modulation in fibromyalgia: A meta-analysis of neuroimaging studies. Neurosci. Biobehav. Rev..

[28] Napadow V., Harris R.E. (2014). What has functional connectivity and chemical neuroimaging in fibromyalgia taught us about the mechanisms and management of ‘centralized’ pain?. Arthritis Res. Ther..

[29] Fallon N. (2018). Altered brain network connectivity during experimental pain in fibromyalgia patients: A functional MRI study. PLoS ONE.

[30] McCarberg B.H., Peppin J.F. (2019). Pain pathway pharmacology: A review. Pain Med..

[31] Kelleher E.M., Lange F., Wanigasekera V., Rathod-Mistry T., Nichols T., Seymour B., Tracey I., Segerdahl A.R., Irani A. (2025). Brain signatures of nociplastic pain: Fibromyalgia Index and descending modulation at population level. Brain.

[32] Staud R. (2011). Peripheral pain mechanisms in chronic widespread pain. Best Pract. Res. Clin. Rheumatol..

[33] Harris R.E., Sundgren P.C., Craig A.D., Kirshenbaum E., Sen A., Napadow V., Clauw D.J. (2009). Elevated insular glutamate in fibromyalgia is associated with experimental pain intensity. Arthritis Rheum..

[34] García-Domínguez M. (2025). Fibromyalgia and Inflammation: Unrevealing the Connection. Cells.

[35] Latremoliere A., Woolf C.J. (2009). Central sensitization: A generator of pain hypersensitivity by central neural plasticity. J. Pain.

[36] Lian Y.N., Wang Y., Zhang Y., Yang C.X. (2020). Duloxetine for pain in fibromyalgia in adults: A systematic review and a meta-analysis. Int. J. Neurosci..

[37] Filipovic T., Filipović A., Nikolic D., Gimigliano F., Stevanov J., Hrkovic M., Bosanac I. (2025). Fibromyalgia: Understanding, Diagnosis and Modern Approaches to Treatment. J. Clin. Med..

[38] Ceko M., Bushnell M.C., Gracely R.H. (2012). Neurobiology underlying fibromyalgia symptoms. Pain Res. Treat..

[39] Ji R.R., Nackley A., Huh Y., Terrando N., Maixner W. (2018). Neuroinflammation and Central Sensitization in Chronic and Widespread Pain. Anesthesiology.

[40] Ichesco E., Schmidt-Wilcke T., Bhavsar R., Clauw D.J., Peltier S.J., Kim J., Napadow V., Hampson J.P., Kairys A.E., Williams D.A. (2014). Altered resting state connectivity of the insular cortex in individuals with fibromyalgia. J. Pain.

[41] Ceko M., Bushnell M.C., Fitzcharles M.A., Schweinhardt P. (2013). Fibromyalgia interacts with age to change the brain. Neuroimage Clin..

[42] Vecchio E., Lombardi R., Paolini M., Libro G., Delussi M., Ricci K., Quitadamo S.G., Gentile E., Girolamo F., Iannone F. (2020). Peripheral and central nervous system correlates in fibromyalgia. Eur. J. Pain.

[43] Gao Y.J., Ji R.R. (2010). Chemokines, neuronal-glial interactions, and central processing of neuropathic pain. Pharmacol. Ther..

[44] Siracusa R., Paola R.D., Cuzzocrea S., Impellizzeri D. (2021). Fibromyalgia: Pathogenesis, Mechanisms, Diagnosis and Treatment Options Update. Int. J. Mol. Sci..

[45] Schweinhardt P., Bushnell M.C. (2010). Pain imaging in health and disease. Trends Neurosci..

[46] Loggia M.L., Kim J., Gollub R.L., Vangel M.G., Kirsch I., Kong J., Wasan A.D., Napadow V. (2013). Default mode network connectivity encodes clinical pain: An arterial spin labeling study. Pain.

[47] Gracely R.H., Petzke F., Wolf J.M., Clauw D.J. (2002). Functional magnetic resonance imaging evidence of augmented pain processing in fibromyalgia. Arthritis Rheum..

[48] Staud R. (2009). Quantitative sensory testing and its relevance in fibromyalgia. Curr. Rheumatol. Rep..

[49] Staud R. (2009). Abnormal pain modulation in patients with spatially distributed chronic pain: Fibromyalgia. Rheum. Dis. Clin. N. Am..

[50] O’Brien A.T., Deitos A., Triñanes Pego Y., Fregni F., Carrillo-de-la-Peña M.T. (2018). Defective Endogenous Pain Modulation in Fibromyalgia: A Meta-Analysis of Temporal Summation and Conditioned Pain Modulation Paradigms. J. Pain.

[51] Staud R., Godfrey M.M., Stroman P.W. (2023). Fibromyalgia is associated with hypersensitivity but not with abnormal pain modulation: Evidence from QST trials and spinal fMRI. Front. Pain Res..

[52] Staud R., Godfrey M.M., Riley J.L., Fillingim R.B. (2023). Efficiency of pain inhibition and facilitation of fibromyalgia patients is not different from healthy controls: Relevance of sensitivity-adjusted test stimuli. Br. J. Pain.

[53] Hubbard C.S., Lazaridou A., Cahalan C.M., Kim J., Edwards R.R., Napadow V., Loggia M.L. (2020). Aberrant Salience? Brain Hyperactivation in Response to Pain Onset and Offset in Fibromyalgia. Arthritis Rheumatol..

[54] Staud R., Godfrey M.M., Robinson M.E. (2021). Fibromyalgia Patients Are Not Only Hypersensitive to Painful Stimuli But Also to Acoustic Stimuli. J. Pain.

[55] Seminowicz D.A., Shpaner M., Keaser M.L., Krauthamer G.M., Mantegna J., Dumas J.A., Newhouse P.A., Filippi C.G., Keefe F.J., Naylor M.R. (2013). Cognitive-behavioral therapy increases prefrontal cortex gray matter in patients with chronic pain. J. Pain.

[56] Heinricher M.M., Tavares I., Leith J., Lumb B. (2009). Descending control of nociception: Specificity, recruitment and plasticity. Brain Res. Rev..

[57] Fields H.L., Basbaum A.I., McMahon S.B., Koltzenburg M., Tracey I., Turk D.C. (2006). Central nervous system mechanisms of pain modulation. Wall and Melzack’s Textbook of Pain.

[58] Aston-Jones G., Cohen J.D. (2005). An integrative theory of locus coeruleus-norepinephrine function: Adaptive gain and optimal performance. Annu. Rev. Neurosci..

[59] Ossipov M.H., Morimura K., Porreca F. (2014). Descending pain modulation and chronification of pain. Curr. Opin. Support. Palliat. Care.

[60] Gil-Ugidos A., Vázquez-Millán A., Samartin-Veiga N., Carrillo-de-la-Peña M.T. (2024). Conditioned pain modulation (CPM) paradigm type affects its sensitivity as a biomarker of fibromyalgia. Sci. Rep..

[61] Staud R., Domingo M. (2003). Evidence for abnormal pain processing in fibromyalgia syndrome. Curr. Rheumatol. Rep..

[62] Yarnitsky D. (2010). Conditioned pain modulation (the diffuse noxious inhibitory control-like effect): Its relevance for acute and chronic pain states. Curr. Opin. Anaesthesiol..

[63] Le Bars D., Dickenson A.H., Besson J.M. (1979). Diffuse noxious inhibitory controls (DNIC). II. Lack of effect on non-convergent neurones, supraspinal involvement and theoretical implications. Pain.

[64] Potvin S., Larouche A., Normand E., de Souza J.B., Gaumond I., Marchand S., Grignon S. (2010). No relationship between the ins del polymorphism of the serotonin transporter promoter and pain perception in fibromyalgia patients and healthy controls. Eur. J. Pain.

[65] Caumo W., Deitos A., Carvalho S., Leite J., Carvalho F., Dussán-Sarria J.A., Lopes Tarragó Mda G., Souza A., Torres I.L., Fregni F. (2016). Motor Cortex Excitability and BDNF Levels in Chronic Musculoskeletal Pain According to Structural Pathology. Front. Hum. Neurosci..

[66] Hilgenberg-Sydney P.B., Kowacs P.A., Conti P.C. (2016). Somatosensory evaluation in Dysfunctional Syndrome patients. J. Oral Rehabil..

[67] Potvin S., Larouche A., Normand E., de Souza J.B., Gaumond I., Grignon S., Marchand S. (2009). DRD3 Ser9Gly polymorphism is related to thermal pain perception and modulation in chronic widespread pain patients and healthy controls. J. Pain.

[68] Jensen K.B., Kosek E., Petzke F., Carville S., Fransson P., Marcus H., Williams S.C., Choy E., Giesecke T., Mainguy Y. (2012). Evidence of dysfunctional pain inhibition in fibromyalgia: Altered fMRI responses to conditioning stimuli. Arthritis Rheum..

[69] Napadow V., LaCount L., Park K., As-Sanie S., Clauw D.J., Harris R.E. (2010). Intrinsic brain connectivity in fibromyalgia is associated with chronic pain intensity. Arthritis Rheum..

[70] Becker S., Schweinhardt P. (2012). Dysfunctional neurotransmitter systems in fibromyalgia, their role in central stress circuitry and pharmacological actions on these systems. Pain Res. Treat..

[71] Naylor J.C. (2021). Norepinephrine and serotonin in chronic pain. Curr. Opin. Neurobiol..

[72] Moont R. (2013). Deficient serotonin activity and increased pain sensitivity in fibromyalgia. J. Pain Res..

[73] Meeus M., Nijs J. (2007). Central sensitization: A biopsychosocial explanation for chronic widespread pain in fibromyalgia and chronic fatigue syndrome. Clin. Rheumatol..

[74] Wood P.B. (2008). Role of central dopamine in pain and analgesia. Expert Rev. Neurother..

[75] Russell I.J. (1994). Elevated cerebrospinal fluid levels of substance P in patients with the fibromyalgia syndrome. Arthritis Rheum..

[76] Arnold L.M. (2013). Pharmacotherapy for fibromyalgia. Expert Rev. Neurother..

[77] Truini A., Tinelli E., Gerardi M.C., Calistri V., Iannuccelli C., La Cesa S., Tarsitani L., Mainero C., Sarzi-Puttini P., Cruccu G. (2016). Abnormal resting state functional connectivity of the periaqueductal grey in patients with fibromyalgia. Clin. Exp. Rheumatol..

[78] Fallon N., Chiu Y., Nurmikko T., Stancak A. (2016). Functional Connectivity with the Default Mode Network Is Altered in Fibromyalgia Patients. PLoS ONE.

[79] Ji R.R., Xu Z.Z., Gao Y.J. (2014). Emerging targets in neuroinflammation-driven chronic pain. Nat. Rev. Drug Discov..

[80] Findeisen K., Guymer E., Littlejohn G. (2025). Neuroinflammatory and Immunological Aspects of Fibromyalgia. Brain Sci..

[81] Bäckryd E. (2017). Evidence of central inflammation in fibromyalgia—Increased cerebrospinal fluid interleukin-8 levels. J. Neuroimmunol..

[82] Kosek E. (2016). Neuroinflammation in fibromyalgia. Neurosci. Lett..

[83] Niddam D.M. (2020). Elevated inflammatory markers in fibromyalgia syndrome. Clin. Rheumatol..

[84] Martinez-Lavin M. (2017). The role of neuroinflammation in fibromyalgia. Curr. Rheumatol. Rep..

[85] Mueller C., Fang Y.D., Jones C., McConathy J.E., Raman F., Lapi S.E., Younger J.W. (2023). Evidence of neuroinflammation in fibromyalgia syndrome: A [18F]DPA-714 positron emission tomography study. Pain.

[86] Albrecht D.S., Forsberg A., Sandström A., Bergan C., Kadetoff D., Protsenko E., Lampa J., Lee Y.C., Höglund C.O., Catana C. (2019). Brain glial activation in fibromyalgia—A multi-site positron emission tomography investigation. Brain Behav. Immun..

[87] Schmidt-Wilcke T. (2017). Neuroinflammation in fibromyalgia. J. Pain Res..

[88] Younger J. (2013). Low-dose naltrexone for fibromyalgia: A randomized controlled trial. Arthritis Rheum..

[89] Burgmer M. (2009). Structural brain alterations in fibromyalgia: A voxel-based morphometry study. Pain.

[90] Lutz J. (2008). Fibromyalgia syndrome: Gray matter decrease in the pain-matrix. Brain.

[91] Kuchinad A., Schweinhardt P., Seminowicz D.A., Wood P.B., Chizh B.A., Bushnell M.C. (2007). Accelerated brain gray matter loss in fibromyalgia patients: Premature aging of the brain?. J. Neurosci..

[92] Kim J. (2015). White matter abnormalities in fibromyalgia: A diffusion tensor imaging study. Pain.

[93] Cagnie B. (2014). Central sensitization in fibromyalgia: A systematic review on structural and functional brain MRI. Semin. Arthritis Rheum..

[94] Sandström A., Ellerbrock I., Tour J., Kadetoff D., Jensen K., Kosek E. (2023). Dysfunctional Activation of the Dorsolateral Prefrontal Cortex During Pain Anticipation Is Associated With Altered Subsequent Pain Experience in Fibromyalgia Patients. J. Pain.

[95] Jensen K.B. (2013). Altered brain connectivity in fibromyalgia correlates with cognitive symptoms. Pain.

[96] Pomares F.B., Funck T., Feier N.A., Roy S., Daigle-Martel A., Ceko M., Narayanan S., Araujo D., Thiel A., Stikov N. (2017). Histological Underpinnings of Grey Matter Changes in Fibromyalgia Investigated Using Multimodal Brain Imaging. J. Neurosci..

[97] Favretti M., Iannuccelli C., Di Franco M. (2023). Pain Biomarkers in Fibromyalgia Syndrome: Current Understanding and Future Directions. Int. J. Mol. Sci..

[98] Tapia-Haro R.M., Molina F., Rus A., Casas-Barragán A., Correa-Rodríguez M., Aguilar-Ferrándiz M.E. (2023). Serum VEGF and CGRP Biomarkers: Relationships with Pain Intensity, Electric Pain, Pressure Pain Threshold, and Clinical Symptoms in Fibromyalgia—An Observational Study. Int. J. Mol. Sci..

[99] Ahmad B., Barkana B.D. (2025). Pain and the Brain: A Systematic Review of Methods, EEG Biomarkers, Limitations, and Future Directions. Neurol. Int..

[100] Cambay V.Y., Hafeez Baig A., Aydemir E., Tuncer T., Dogan S. (2024). Minimum and Maximum Pattern-Based Self-Organized Feature Engineering: Fibromyalgia Detection Using Electrocardiogram Signals. Diagnostics.

[101] Potvin S., Marchand S. (2016). Pain facilitation and pain inhibition during conditioned pain modulation in fibromyalgia and in healthy controls. Pain.

[102] Kosek E., Cohen M., Baron R., Gebhart G.F., Mico J.A., Rice A.S.C., Rief W., Sluka A.K. (2016). Do we need a third mechanistic descriptor for chronic pain states?. Pain.

[103] Lewis G.N., Heales L., Rice D.A., Rome K., McNair P.J. (2012). Reliability of the conditioned pain modulation paradigm to assess endogenous inhibitory pain pathways. Pain Res. Manag..

[104] Flodin P., Martinsen S., Löfgren M., Bileviciute-Ljungar I., Kosek E., Fransson P. (2014). Fibromyalgia is associated with decreased connectivity between pain- and sensorimotor brain areas. Brain Connect.

[105] Loggia M.L., Chonde D.B., Akeju O., Arabasz G., Catana C., Edwards R.R., Hill E., Hsu S., Izquierdo-Garcia D., Ji R.R. (2015). Evidence for brain glial activation in chronic pain patients. Brain.

[106] Bazzichi L., Rossi A., Massimetti G., Giannaccini G., Giuliano T., De Feo F., Ciapparelli A., Dell’Osso L., Bombardieri S. (2007). Cytokine patterns in fibromyalgia and their correlation with clinical manifestations. Clin. Exp. Rheumatol..

[107] O’Mahony L.F., Srivastava A., Mehta P., Ciurtin C. (2021). Is fibromyalgia associated with a unique cytokine profile? A systematic review and meta-analysis. Rheumatology.

[108] Haas L., Portela L.V., Böhmer A.E., Oses J.P., Lara D.R. (2010). Increased plasma levels of brain derived neurotrophic factor (BDNF) in patients with fibromyalgia. Neurochem. Res..

[109] Uçeyler N., Valenza R., Stock M., Schedel R., Sprotte G., Sommer C. (2006). Reduced levels of antiinflammatory cytokines in patients with chronic widespread pain. Arthritis Rheum..

[110] Fayed N., Garcia-Campayo J., Magallón R., Andrés-Bergareche H., Luciano J.V., Andres E., Beltrán J. (2010). Localized 1H-NMR spectroscopy in patients with fibromyalgia: A controlled study of changes in cerebral glutamate/glutamine, inositol, choline, and N-acetylaspartate. Arthritis Res. Ther..

[111] Feraco P., Bacci A., Pedrabissi F., Passamonti L., Zampogna G., Pedrabissi F., Malavolta N., Leonardi M. (2011). Metabolic abnormalities in pain-processing regions of patients with fibromyalgia: A 3T MR spectroscopy study. AJNR Am. J. Neuroradiol..

[112] Menzies V., Lyon D.E., Archer K.J., Zhou Q., Brumelle J., Jones K.H., Gao G., York T.P., Jackson-Cook C. (2013). Epigenetic alterations and an increased frequency of micronuclei in women with fibromyalgia. Nurs. Res. Pract..

[113] Bjersing J.L., Bokarewa M.I., Mannerkorpi K. (2015). Profile of circulating microRNAs in fibromyalgia and their relation to symptom severity: An exploratory study. Rheumatol. Int..

[114] Tanriverdi F., Karaca Z., Unluhizarci K., Kelestimur F. (2007). The hypothalamo-pituitary-adrenal axis in chronic fatigue syndrome and fibromyalgia syndrome. Stress.

[115] Riva R., Mork P.J., Westgaard R.H., Rø M., Lundberg U. (2010). Fibromyalgia syndrome is associated with hypocortisolism. Int. J. Behav. Med..

[116] Xiao Y., Haynes W.L., Michalek J.E., Russell I.J. (2013). Elevated serum high-sensitivity C-reactive protein levels in fibromyalgia syndrome patients correlate with body mass index, interleukin-6, interleukin-8, erythrocyte sedimentation rate. Rheumatol. Int..

[117] Zetterman T., Markkula R., Kalso E. (2022). Elevated highly sensitive C-reactive protein in fibromyalgia associates with symptom severity. Rheumatol. Adv. Pract..

[118] Sanada K., Díez M.A., Valero M.S., Pérez-Yus M.C., Demarzo M.M., García-Toro M., García-Campayo J. (2015). Effects of non-pharmacological interventions on inflammatory biomarker expression in patients with fibromyalgia: A systematic review. Arthritis Res. Ther..

[119] Arnold L.M. (2004). Efficacy of duloxetine in patients with fibromyalgia: A randomized controlled trial. JAMA.

[120] Goldenberg D.L. (2004). Pharmacologic treatment of fibromyalgia syndrome. JAMA.

[121] Younger J., Mackey S. (2009). Fibromyalgia symptoms are reduced by low-dose naltrexone: A pilot study. Pain Med..

[122] Bruun K.D., Christensen R., Amris K., Blichfeldt-Eckhardt M.R., Bye-Møller L., Henriksen M., Alkjaer T., Toft P., Holsgaard-Larsen A., Vaegter H.B. (2025). Effect of Naltrexone on Spinal and Supraspinal Pain Mechanisms and Functional Capacity in Women with Fibromyalgia: Exploratory Outcomes from the Randomized Placebo-Controlled FINAL Trial. CNS Drugs.

[123] Häuser W. (2010). Efficacy of exercise in patients with fibromyalgia syndrome: A systematic review and meta-analysis of randomized controlled trials. Arthritis Res. Ther..

[124] Thieme K. (2006). Cognitive-behavioral therapy for fibromyalgia syndrome: A systematic review and meta-analysis of randomized controlled trials. Arthritis Rheum..

[125] Mhalla A. (2010). Repetitive transcranial magnetic stimulation in fibromyalgia: A randomized controlled trial. Brain.

[126] Silva V.A., Baptista A.F., Fonseca A.S., Carneiro A.M., Brunoni A.R., Carrilho P.E.M., Lins C.C., Kubota G.T., Fernandes A., Lapa J.D.S. (2025). Motor cortex repetitive transcranial magnetic stimulation in fibromyalgia: A multicentre randomised controlled trial. Br. J. Anaesth..

[127] Velickovic Z., Radunovic G. (2024). Repetitive Transcranial Magnetic Stimulation in Fibromyalgia: Exploring the Necessity of Neuronavigation for Targeting New Brain Regions. J. Pers. Med..

[128] Fang H., Hou Q., Zhang W., Su Z., Zhang J., Li J., Lin J., Wang Z., Yu X., Yang Y. (2024). Fecal Microbiota Transplantation Improves Clinical Symptoms of Fibromyalgia: An Open-Label, Randomized, Nonplacebo-Controlled Study. J. Pain.

[129] Cai W., Haddad M., Haddad R., Kesten I., Hoffman T., Laan R., Westfall S., Defaye M., Abdullah N.S., Wong C. (2025). The gut microbiota promotes pain in fibromyalgia. Neuron.

[130] Wang Z., Jiang D., Zhang M., Teng Y., Huang Y. (2023). Causal association between gut microbiota and fibromyalgia: A Mendelian randomization study. Front. Microbiol..

[131] Marum A.P., Moreira C., Saraiva F., Tomas-Carus P., Sousa-Guerreiro C. (2016). A low fermentable oligo-di-mono saccharides and polyols (FODMAP) diet reduced pain and improved daily life in fibromyalgia patients. Scand. J. Pain.

[132] Nhu N.T., Chen D.Y., Yang Y.S.H., Lo Y.C., Kang J.H. (2024). Associations Between Brain-Gut Axis and Psychological Distress in Fibromyalgia: A Microbiota and Magnetic Resonance Imaging Study. J. Pain.

[133] Harte S.E. (2018). Biomarkers in fibromyalgia: Present and future directions. Curr. Pain Headache Rep..

[134] Kamaly N.A., Kamel A.S., Sadik N.A., Shahin N.N. (2025). Milnacipran and Vanillin Alleviate Fibromyalgia-Associated Depression in Reserpine-Induced Rat Model: Role of Wnt/β-Catenin Signaling. Mol. Neurobiol..

[135] Arnold L.M., Clauw D.J. (2017). Challenges of implementing fibromyalgia treatment guidelines in current clinical practice. Postgrad. Med..

[136] Macfarlane G.J., Kronisch C., Dean L.E., Atzeni F., Häuser W., Fluß E., Choy E., Kosek E., Amris K., Branco J. (2017). EULAR revised recommendations for the management of fibromyalgia. Ann. Rheum. Dis..

[137] Kundakci B., Hall M., Atzeni F., Branco J., Buskila D., Clauw D., Crofford L.J., Fitzcharles M.A., Georgopoulos V., Gerwin R.D. (2022). International, multidisciplinary Delphi consensus recommendations on non-pharmacological interventions for fibromyalgia. Semin. Arthritis Rheum..

[138] Murphy A.E., Minhas D., Clauw D.J., Lee Y.C. (2023). Identifying and Managing Nociplastic Pain in Individuals With Rheumatic Diseases: A Narrative Review. Arthritis Care Res..

